# A Preliminary Evaluation of ^177^Lu-PSMA-617-Based Targeted Radioligand Therapy with X-Ray Stimulated PSMA Relocation Using the *PiggyBac* Reporter-Gene-Engineered Orthotopic Prostate Tumor Model

**DOI:** 10.3390/pharmaceutics18081009

**Published:** 2026-08-14

**Authors:** Yen-Ta Chen, Ke-Hsin Huang, Chun-Yi Wu, Yi-Jang Lee

**Affiliations:** 1 Division of Urology, Department of Surgery, Kaohsiung Chang Gung Memorial Hospital, Chang Gung University College of Medicine, Kaohsiung 833401, Taiwan; 2 Department of Biomedical Imaging and Radiological Sciences, National Yang-Ming Chiao Tung University, Taipei 112304, Taiwan; 3 Cancer and Immunology Research Center, National Yang Ming Chiao Tung University, Taipei Branch, Taipei 112304, Taiwan

**Keywords:** slow-growing prostate cancer, *PiggyBac* transposon system, reporter gene imaging, prostate-specific membrane antigen, orthotopic tumor model

## Abstract

**Background/Objectives:** Because human prostate cancer (PCa) typically exhibits slow tumor growth, establishing reliable PCa tumor models is often time-consuming and unpredictable, thereby limiting the efficiency of preclinical theranostic research. To overcome this limitation, this study employed a non-viral *PiggyBac* transposon system to introduce triple-reporter genes into PSMA-expressing C4-2 cells, generating orthotopic and subcutaneous xenograft models that allow noninvasive, real-time monitoring of PCa progression and treatment response. **Methods:** Reporter-engineered C4-2 3R cells were generated by co-transfecting constructs encoding the reporter cassette and PB transposase, followed by enrichment through fluorescence microscopy and fluorescence-activated cell sorting (FACS) and implantation orthotopically or subcutaneously into mice. Tumor growth and treatment response to a single 2 Gy X-ray dose followed by 14.8 MBq of ^177^Lu-PSMA-617, or to each monotherapy, were monitored weekly using an IVIS imaging system. Imaging findings were validated by tumor dissection and hematoxylin and eosin (H&E) staining, while PSMA expression was assessed by Western blotting and ^18^F-PSMA-1007 PET/CT. **Results:** C4-2 3R cells stably expressed the triple reporter genes (mRFP, luc2, and HSV1-tk), generating detectable orthotopic bioluminescence within one week and persisting for at least five weeks. In contrast, subcutaneous implantation generated only transient luc2 signals with no tumor formation. X-ray exposure did not increase total PSMA levels but induced the redistribution of PSMA to the cell membrane. Combined external beam radiotherapy (EBRT) and ^177^Lu-PSMA-617 treatment produced higher ^18^F-PSMA-1007 uptake and stronger tumor suppression, with minimal residual tumor mass, as compared to single-treatment or control groups. **Conclusions:** This preliminary investigation suggests that the C4-2 3R model provides a practical and trackable tool for investigating slow-growing PCa tumors and evaluating PSMA-targeted therapies, either alone or in combination with EBRT.

## 1. Introduction

Prostate cancer (PCa) is the most frequently diagnosed malignancy and one of the leading causes of cancer-related death among men worldwide. Although PCa is generally considered a slow-growing and low-grade cancer type, certain subtypes of PCa can progress into aggressive disease and develop into metastatic castration-resistant prostate cancer (mCRPC) [1]. The slow-growing nature of PCa also presents a major challenge in establishing PCa animal models, especially subcutaneous xenograft models, which may require more than two months for tumor formation (volume doubling time is 86 h) [2]. In many cases, measurable tumor growth may not occur even after prolonged observation. Such slow kinetics and unpredictable outcomes limit the throughput of preclinical screening, delay longitudinal imaging studies, and increase animal housing and maintenance costs. Therefore, a real-time, noninvasive approach would be valuable for monitoring the growing potential of human PCa cells in xenograft models and for accurately assessing the efficacy of novel therapeutic strategies.

Recent advances in molecular imaging and targeted radionuclide therapy have significantly improved PCa management. Reporter gene imaging (RGI) refers to the introduction of one or multiple functional genes encoding fluorescent proteins, bioluminescent enzymes or functional proteins into cells of interest, allowing visualization by optical imaging or radionuclide-based imaging such as PET and SPECT [3]. Although retroviral-mediated transduction can provide stable reporter gene expression through chromosomal integration, its application is limited by the potential induction of both intended and unintended immune responses [4,5]. In addition, viral gene delivery requires polybrene, which may cause cytotoxicity during lentiviral transduction [6,7]. In contrast, non-viral gene delivery methods, such as electroporation, liposomes, and cationic polymers, are relatively safer and less immunogenic, but they typically result in transient gene delivery with limited stability [4,5,8,9]. DNA transposon-based gene delivery systems have been developed as an alternative approach that combines the advantages of viral and non-viral delivery. These systems typically utilize a dual-plasmid strategy consisting of a transposase expression vector and a gene of interest flanked by inverted terminal repeats (ITRs) [10,11]. Among currently available transposon systems, the *PiggyBac* transposon system exhibits high transposition activity and low overproduction inhibition compared with other transposon systems in mammalian cells [12,13]. Previous studies have demonstrated that the *PiggyBac* system can establish stable cell lines expressing multiple reporter genes in murine breast cancer cells and human hypopharyngeal cancer cells. Although transfection efficiencies in these cell types are relatively low, transfected cells can be enriched by flow cytometry and maintain long-term reporter gene expression [14,15]. For monitoring the progression and therapeutic responses of slow-growing cancers such as PCa, RGI requires a convenient and cost-effective approach suitable for long-term use and repeated evaluation of tumor growth in vivo, especially in orthotopic tumor models. Therefore, the *PiggyBac* transposon system may represent a promising strategy for establishing stable reporter-expressing PCa cells for long-term preclinical investigation due to tis low immunogenicity potential and stable reporter gene expression.

Prostate-specific membrane antigen (PSMA) has emerged as an important molecular target for both diagnosis and therapy. ^68^Ga-PSMA PET/CT provides sensitive detection of recurrent and metastatic lesions, while ^177^Lu-PSMA-617 has demonstrated significant improvements in overall survival among patients with mCRPC, and has been approved by the US Food and Drug Administration (FDA) and European Medicines Agency [16,17]. Despite these clinical advances, further translation of novel theranostic strategies remains limited by the lack of robust preclinical models. In particular, combination treatment with external beam radiotherapy (EBRT) or immunotherapy with ^177^Lu-PSMA-617 has been considered a promising approach for PCa treatment; however, robust and reliable small-animal models remain essential for preclinical validation and translational investigation [18,19,20]. Most murine tumor models used to evaluate the efficacy of ^177^Lu-PSMA-617 monotherapy or combination therapy rely on subcutaneous xenograft models established using PSMA-positive LNCaP cells or PSMA transduced PC3 cells (PC3-PIP), both of which exhibit relatively slow tumor growth kinetics [19,21,22,23]. In contrast, prostate-directed orthotopic models are less frequently reported in related studies, despite their potential to more accurately reproduce the tumor microenvironment and tumor–stromal interaction associated with PCa progression [23]. However, the technical challenges associated with anatomic engrafting and real-time monitoring of tumor progression remain major limitations for the application of this important preclinical model.

In this study, we introduced a *PiggyBac* transposon system carrying triple reporter genes, including an improved luciferase gene (*luc2*), a red fluorescence protein gene (*RFP*), and a herpes simplex virus type I thymidine kinase gene (*HSV1-tk*), into human C4-2 PCa cells, which were derived from a subcutaneous LNCaP xenograft tumor. The established C4-2 3R cells were compared for their tumor-forming capacity in subcutaneous xenograft and prostate orthotopic models using bioluminescence imaging. Moreover, the C4-2 3R orthotopic tumor model was applied to assess tumor responses following single treatment with ^177^Lu-PSMA-617, X-ray irradiation, or a combination of both regimens. Our findings suggest that C4-2 3R cells exhibited robust growth kinetics in the orthotopic model, and can be readily tracked for in vivo evaluation of tumor responses following different therapeutic approaches.

## 2. Materials and Methods

### 2.1. Cell Culture

Human PCa C4-2 cells were purchased from American Type Culture Collection (ATCC, Cat# CRL-3314, Manassas, VA, USA). Cells were cultured in Roswell Park Memorial Institute (RPMI) 1640 medium (Gibco^®^ ThermoFisher Scientific, Waltham, MA, USA) supplemented with 10% fetal bovine serum (FBS, HyClone^®^ ThermoFisher Scientific, Waltham, MA, USA), 1% penicillin-streptomycin solution (100×) (Gibco^®^ ThermoFisher Scientific, Waltham, MA, USA) and 1% L-glutamine (200 mM) (Sigma-Aldrich Co., St. Louis, MO, USA). Cells were maintained in 37 °C incubator filled with 5% CO_2_ in air and were passaged every two days. The C4-2 3R cells were cultured under the same conditions after stable cell lines were established.

### 2.2. Preparation of Radiopharmaceuticals

PSMA-617 was obtained from MedChemExpress USA (MCE LLC, Cat# HY-117410, Monmouth Junction, NJ, USA). The ^177^Lu-PSMA-617 was prepared according to the protocol described below. First, PSMA-617 (10 mM in DMSO), sodium acetate (0.4 M, pH 5.5), 20% ascorbic acid solution, and approximately 185-370 MBq of ^177^Lu-LuCl_3_ (Isotopia Nuclear Medicine, Petach Tikva, Israel) were combined in a reaction vial, and incubated at 95 °C for 60 min. Labeling efficiency was assessed by a radio-thin layer chromatography (radio-TLC, AR2000, Bioscan, Washington, DC, USA) system using instant thin-layer chromatography (ITLC) plates (Merck, Darmstadt, Germany) as the stationary phase and 0.5 M sodium citrate buffer (pH = 5.0) as the mobile phase. After the reaction, the crude product was loaded onto a C18 Sep-Pak cartridge, which was preconditioned with 10 mL of ethanol, followed by 10 mL of ddH_2_O. The desired compound was eluted with acetonitrile/water solution (1:1, *v*/*v*), evaporated to dryness under reduced pressure with a nitrogen stream, and reconstituted in sterile normal saline for subsequent experiments. The radiochemical purity of ^177^Lu-PSMA-617, as determined by high-performance liquid chromatography (HPLC), exceeded 95% (Supplementary Figure S1). The non-decay-corrected radiochemical yield was 72 ± 6%, and the molar activity was approximately 47 GBq/μmol. ^18^F-PSMA-1007 was purchased from the Department of Nuclear Medicine, Taipei Veteran General Hospital.

### 2.3. Transfection of PiggyBac Transposon Constructs and Cell Sorting

The *PiggyBac* transposon plasmid PB-3R-puro was constructed as reported before [24]. This plasmid was co-transfected with Act-PBase plasmid [25] into C4-2 cells using PolyJet^TM^ reagent (SignaGen, Frederick, MD, USA) by following the manufacturer’s protocol. A plasmid ratio of 4:1 (PB-3R-puro and Act-PBase) was used, with 5 μg total plasmids for transfection. Forty-eight hours after transfection, mRFP expressing cells were identified by fluorescence microscopy (CKX53, Olympus, Hachioji, Japan). The transfected C4-2 cells were then sorted using the flow cytometry (CytoFLEX SRT, Beckman Coulter, Brea, CA, USA). Briefly, 1 × 10^7^ cells were resuspended in the fluorescence activated cell sorting (FACS) buffer (1% FBS in phosphate-buffered solution buffer) and injected into the flow cytometer to sort out the red fluorescent cells for enrichment. The enriched cells were routinely passaged and visualized using the fluorescent microscope to confirm the expression of mRFP reporter protein.

### 2.4. Luciferase Reporter Gene Assay

For luciferase activity measurement, 1 × 10^5^ cells were seeded into each well of a 96-well plate (Cat# 655083, CELLSTAR^®^, Greiner Bio-One, Kremsmünster, Austria) in 100 μL of RPMI 1640 medium, with five replicate wells per group. Each well was mixed with 100 μL of D-luciferin solution (15 mg/mL, Cat# LUCK-1G, GOLDBIO, St. Louis, MO, USA), and measured using the ELISA reader (Infinite^®^ 200 PRO, TECAN, Seestrasse, Männedorf, Switzerland).

### 2.5. Cell Viability Assay

Cell viability was evaluated using the MTT assay. Briefly, 1 × 10^4^ cells were seeded into each well of a 96-well plate, and treated with increasing concentrations of ganciclovir (GCV; 0–40 μM; Sigma-Aldrich Co., St. Louis, MO, USA) for four days. Cells were then incubated with 1 mg/mL MTT solution (3-[4,5-dimethylthiazol-2-yl]-2,5-diphenylterazoliumbromide, Sigma-Aldrich Co., St. Louis, MO, USA) in serum-free medium for 3 h. After removal of the MTT solution, the formed crystals were dissolved in 100 μL of dimethyl sulfoxide (DMSO). The plate was placed in an ELISA reader (Sunrise, TECAN Group Ltd., Männedorf, Switzerland) and scanned by 570 nm of absorbed wavelength.

### 2.6. X-Rays Source

Radiation exposure was performed using a cabinet X-rays irradiator (X-Rad 225XL, Precision, Madison, CT, USA) at a dose rate of 134.3 cGy/min.

### 2.7. Establishment of Tumor Models in Small Animals

Five-week-old male Balb/C nu/nu mice (BALB/cAnN.Cg-Foxn1^nu^/CrlNarl) were purchased from the National Laboratory Animal Center (NLAC, Nankang, Taiwan). Mice were acclimatized for one week before tumor inoculation. For orthotopic tumor model, C4-2 3R cells (4.5 × 10^5^) were resuspended in a 1:1 mixture of Matrigel^®^ (Cat# 354248, Corning, Glendale, AZ, USA) and OPTI-MEM (Gibco^®^ ThermoFisher Scientific, Waltham, MA, USA), with a final volume of 20 μL, and gently injected into the surgically exposed prostate as previously reported [26]. BD insulin syringes (1 mL) fitted with 29G needles were used for cell injection (Becton, Dickinson and Company, Franklin Lakes, NJ, USA). Briefly, mice were anesthetized with 3% isoflurane, and sterile surgical instruments were used throughout the procedure. The lower abdomen was disinfected with 70% ethanol followed by povidone–iodine. A midline incision of approximately 1 cm was made in the lower abdomen. The seminal vesicles and bladder were gently exteriorized using a sterile cotton swab to locate the prostate. Following injection, the muscle layer was closed using sterile absorbable 6-0 chromic gut sutures (Cat# CC126, UNIK, New Taipei, Taiwan), and the skin was closed with sterile non-absorbable 4-0 nylon sutures (Cat# NC124, UNIK). The incision site was disinfected with povidone–iodine, and mice were monitored daily for one week to assess wound healing. For the subcutaneous xenograft tumor model, 1 × 10^7^ cells were resuspended in 100 μL of Matrigel^®^ and OPTI-MEM mixture, and subcutaneously injected into the right thigh of each mouse under anesthesia. All animal cares and experiments were approved by the Institutional Animal Care and Use Committee of National Yang Ming Chiao Tung University (Approval IACUC number: 1121202).

### 2.8. Bioluminescence Imaging

Tumor-bearing mice (N = 8 for tumor growth tracking, N = 12 for therapeutic evaluation) were intraperitoneally injected with 150 mg/kg D-luciferin (Caliper Co., Hopkinton, MA, USA) and anesthetized with 2% isoflurane during image acquisition. Mice were then placed in an IVIS Lumina X5 (PerkinElmer, Life and Analytical Sciences Inc., Waltham, MA, USA) to acquire luminescent signals. For quantitative analysis, regions of interest (ROIs) of identical size were applied to the tumor region of each mouse. Total photon fluxes were semi-quantified as photons/sec/cm^2^/sr using the bundled Living Imaging^®^ software (ver. 4.7.4, Revvity, Waltham, MA, USA).

### 2.9. Micro-Magnetic Resonance Imaging (μMRI) for Small Animals

MRI was performed using a 7T PET/MR system (Bruker, Rheinstetten, Germany). A T2-weighted TurboRARE sequence was used to monitor the location of prostate tumor five weeks after tumor implantation. Tumor-bearing mice were anesthetized with 3% isoflurane. The depth of anesthesia, pulse rate, and respiration were continuously monitored throughout the imaging procedure. The sequence parameters are as follows: echo time (TE) = 27.82 ms, repetition time (TR) = 2177 ms, echo train length (ETL) = 8, average = 8, slice thickness = 1 mm, field of view = 35 × 35 mm, matrix size = 192 × 192, in-plane resolution = 156 × 156 µm, 30 slices, and acquisition time = 7 min.

### 2.10. Preparation of Membrane Proteins

The Subcellular Protein Fractionation Kit for Cultured Cells (ThermoFisher Scientific, Waltham, MA, USA) was used to separate membrane proteins and cytoplasmic proteins according to the manufacturer’s instructions.

### 2.11. Western Blot Analysis

Cell lysates were extracted using protein lysis buffer (50 mM Tris-HCl, 120 mM NaCl, 0.5% NP-40) supplemented with 2% proteinase inhibitor (Sigma-Aldrich Co., St. Louis, MO, USA). Samples were mixed at 4 °C for 15 min and then centrifuged for 15 min. Protein concentrations were quantified using the Bio-Rad Protein Assay reagent (Bio-Rad, Bio-Rad Laboratories Inc., Hercules, CA, USA). For protein separation, samples were boiled in sampling buffer [250 mM Tris-HCl pH 6.8, 10% sodium dodecyl sulfate (SDS), 30% glycerol, 5% β-mercaptoethanol, and 0.02% bromophenol blue], and loaded onto 10% SDS-polyacrylamide gel electrophoresis (SDS-PAGE) gels. Electrophoresis was performed at 90 V for 100–120 min. Proteins were then electro-transferred onto nitrocellulous membranes (BioTrace^TM^ NT, Pall, Port Washington, NY, USA). The membranes were blocked with TBST buffer (150 mM NaCl, 10 mM Tris-HCl, 0.1% Tween 20, pH 8.0) containing 4% skim milk. The membranes were incubated with primary antibodies, including anti-PSMA (Cat# 12072, Cell Signaling Technology Inc., Beverly, MA, USA), anti-Na^+^/K^+^ ATPase (Cat# ab76020, Abcam, Cambridge, MA, USA), anti-α-tubulin (Cat# GTX112141, GeneTex Inc., Alton Pkwy Irvine, CA, USA), and anti-glyceraldehyde 3-phosphate dehydrogenase (GAPDH, Cat# MA5-15738, Invitrogen Inc. Carlsbad, CA, USA). Horseradish peroxidase (HRP)-conjugated anti-rabbit or anti-mouse IgG (Millipore Co., Billerica, MA, USA) was used as the secondary antibody. The membrane was rinsed with Trident femto Western HRP substrate (Genetex Inc., Irvine, CA, USA) and chemoluminescent signals were detected using the ImageQuant™ LAS-4000 (GE Healthcare Bio-Science AB, Uppsala, Sweden). Protein band intensity was quantified by densitometric analysis using ImageJ software (Version 1.47).

### 2.12. Flow Cytometric Analysis of PSMA

Cells (1.2 × 10^6^) were cultured for 24 h, and then exposed to different doses of X-rays. After an additional 4 h of incubation, cells were collected in FACS buffer. Following centrifugation, cell pellets were incubated with Alexa Fluor^®^ 488 anti-human PSMA antibody (IgG isotype, Cat# 342506, Biolegend, San Diego, CA, USA) diluted 1:100 in FACS buffer on ice for 1 h. Cells were then centrifuged and resuspended in 500 μL of FACS buffer, and passed through a 37 μm cell strainer. The samples were analyzed using a flow cytometer (CytoFlex, Beckman Coulter, Brea, CA, USA) to detect the fluorescent signals, and the results were normalized to 0 Gy.

### 2.13. Immunofluorescence Microscopy

Cells seeded on coverslips were incubated with PBS-diluted Lipophilic Tracers-DiI (Cat# D282, Thermo Fisher Scientific, Waltham, MA, USA) to stain the cell membrane. The stained cells were then fixed with 4% paraformaldehyde for 10 min, and blocked with 5% bovine serum albumin (BSA) for 1 h. Alexa Fluor^®^ 488 anti-human PSMA antibody (IgG isotype, Cat# 342506, Biolegend, San Diego, CA, USA) was diluted 1:100 in 5% BSA and incubated with cells on coverslips for 12 h. Cell nuclei were stained with 2 μg/mL 4′,6-diamidino-2-phenylindole (DAPI). The coverslips were mounted and sealed on the slide. The images were acquired using the confocal microscope and analyzed using the bundled ZEN3.1 software (ZEISS LSM 880, Oberkochen, Germany).

### 2.14. Radiation Exposure and Radioligand Therapy on Orthotopic Tumors

For X-ray irradiation, tumor-bearing mice (N = 3 for untreated control and each experimental group) were anesthetized with 2% isoflurane and placed in the supine position on the irradiation platform to receive a 2 Gy dose using a cabinet X-ray irradiator. The lower abdomen was centered within the irradiation field, while the remaining body regions were shielded with lead blocks to minimize radiation exposure to normal tissues. For PSMA-targeted radioligand therapy, mice received a single intravenous injection of 14.8 MBq of ^177^Lu-PSMA-617 via the tail vein according to a previous report with slight modification [27]. For the combined treatment, mice received the same dose of ^177^Lu-PSMA-617 4 h after 2 Gy X-ray irradiation to evaluate the effect of radiation pre-treatment on therapeutic efficacy. Body weight was measured every two days to monitor treatment-related toxicity and overall health status.

### 2.15. Positron Emission Tomography/Computed Tomography (PET/CT) for Small Animals

PSMA expression in tumor-bearing mice receiving different treatments was evaluated using a small-animal PET/CT scanner (Mediso nanoScan^®^ PET/CT, Budapest, Hungary) at the Molecular Translational Imaging Center in Chang-Gung Memorial Hospital, Linkou, Taiwan. Mice were administered a single intravenous injection of 11.1 MBq of ^18^F-PSMA-1007 via the tail vein. One hour after tracer administration, PET/CT imaging was performed for 15 min under 2% isoflurane anesthesia. The acquired images were quantified using the volume of interest (VOI) tool in Pmod software (Pmod Version 4.4, Bruker, Billerica, MA, USA). Standardized uptake values (SUVs) were calculated based on the tumor radioactivity concentration, injected dose, and body weight of each mouse.

### 2.16. Hematoxylin and Eosin (H&E) Staining

Tumor tissues were harvested, briefly rinsed with PBS, and fixed in 10% neutral-buffered formalin for 24 h at room temperature. After fixation, samples were dehydrated through a graded ethanol series, cleared in xylene, and embedded in paraffin. Paraffin blocks were sectioned at a thickness of 3 µm using a rotary microtome and mounted onto glass slides. For H&E staining, sections were deparaffinized in xylene and rehydrated through graded ethanol solutions (100%, 95%, 75%; 3 min each) to distilled water. Slides were stained with hematoxylin for nuclear visualization, rinsed with running tap water, and differentiated in acid alcohol when necessary. After bluing in alkaline solution, sections were counterstained with eosin. Slides were then dehydrated through increasing ethanol concentrations, cleared in xylene, and coverslipped using a synthetic resin mounting medium. Stained sections were examined and imaged using a bright-field microscope equipped with a digital camera.

### 2.17. Statistical Analysis

The statistical analysis for cell experiments was performed using Student’s *t*-test and one-way analysis of variance (ANOVA). For multiple-group comparisons, data are presented as the mean ± standard error of the mean (SEM). Because of the small group size in the animal studies, the nonparametric one-way ANOVA (Kruskal–Wallis test) was used for cross-sectional comparisons, whereas two-way ANOVA followed by Tukey’s multiple comparison test was applied to analyze the statistical significance. Graphs were generated using Prism v10.1 (GraphPad Software DBA Statistical Solutions, Franklin St FL Boston, MA, USA).

## 3. Results

### 3.1. Establishment and Validation of PiggyBac Transposon System Mediated Delivery of Triple Reporter Genes into C4-2 Cells

**Figure 1:**
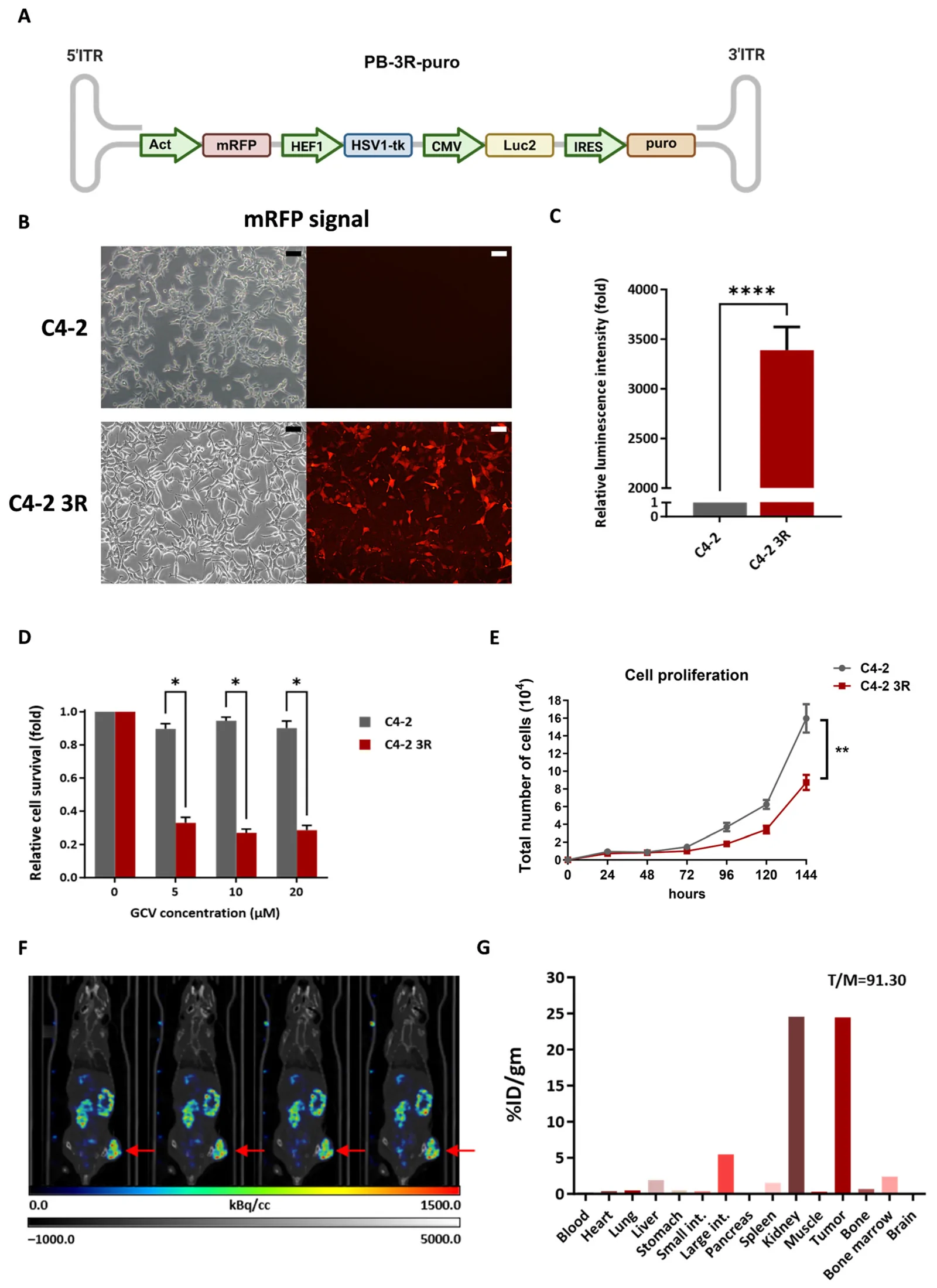
Establishment of C4-2 3R cells. (**A**) Triple reporter genes cassette arranged in the PB-3R-puro construct; (**B**) C4-2 cells and C4-2 3R cells using the fluorescence microscope; (**C**) luciferase activity using the luciferase assay; (**D**) assessing GCV toxicity in HSV1-tk reporter gene expressing cells using the MTT assay; (**E**) comparing cell proliferation between C4-2 cells and C4-2 3R cells; (**F**) four consecutive slices of SPECT/CT imaging for detection of ^177^Lu-PSMA-617 uptake in a C4-2 xenograft tumor; the arrows indicate the locations of tumor uptake; (**G**) biodistribution of ^177^Lu-PSMA-617 in different tissues of this tumor bearing mouse. Bar colors represented corresponding organs. *: *p* < 0.05; **: *p* < 0.01; ****: *p* < 0.0001.

The arrangement of triple reporter genes together with a puromycin resistance cassette flanked by 5’ and 3’ internal terminal repeat (ITR) in the *PiggyBac* construct is illustrated in Figure 1A. C4-2 cells were transfected with the *PiggyBac* construct as described in the Materials and Methods section. The transfection efficiency of C4-2 cells was approximately 7%, as determined by fluorescent microscopy and flow cytometry. mRFP expressing cells were then sorted by FACS and enriched through sub-culture (Supplementary Figure S2). Although C4-2 3R cells were not selected with puromycin, they maintained stable mRFP expression after routine passaging compared with parental C4-2 cells (Figure 1B). The expressions of *luc2* and *HSV1-tk* reporter genes in C4-2 3R cells were further confirmed by luciferase and MTT assays, respectively. C4-2 3R cells showed robust luciferase activity (Figure 1C). These cells also exhibited significant sensitivity to 5–20 µM GCV, a nucleotide analog that is phosphorylated by HSV1-tk and interferes DNA synthesis (Figure 1D). Although C4-2 3R cells proliferated more slowly than parental C4-2 cells, their doubling time was approximately 21 h (Figure 1E). Prior to investigating the tumor-forming capacity of C4-2 3R cells in vivo, parental C4-2 cells were first inoculated subcutaneously into five nude mice to evaluate their tumor formation rate. Only one of the five mice developed a palpable tumor after three months of observation. The tumor-bearing mouse then underwent single photon emission computed tomography (SPECT/CT) imaging following intravenous injection of ^177^Lu-PSMA-617. The C4-2 xenograft showed apparent uptake of ^177^Lu-PSMA-617 (Figure 1F). The mouse was then sacrificed for biodistribution analysis, and the results showed that the tumor and kidney exhibited the highest radioactivity, with a tumor-to-muscle (T/M) ratio of 91.30 (Figure 1G). This result suggests that C4-2 xenograft tumors are targetable by ^177^Lu-PSMA-617, but the rate of subcutaneous tumor formation was low, consistent with previous reports [2,28,29]. For comparison, LNCaP cells were inoculated into nude mice using the same procedure; however, no palpable or visible tumor developed after three months of observation.

### 3.2. RGI of Tumor Formation in Orthotopic Model and Subcutaneous Xenograft Model Using C4-2 3R Cells

**Figure 2:**
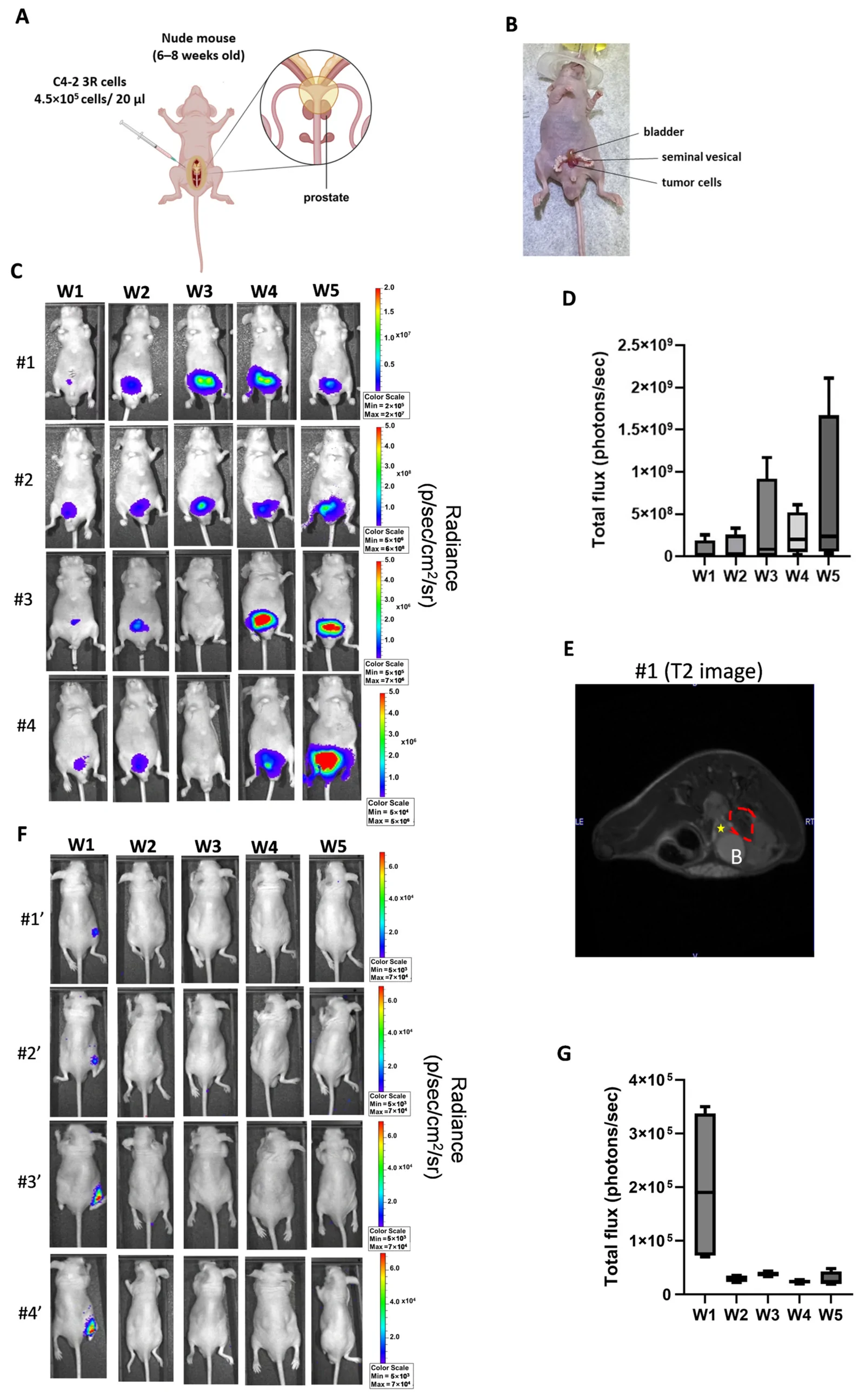
Establishment of PCa tumor models for monitoring of tumor progression by bioluminescent imaging. (**A**) C4-2 3R cells inoculation into prostate; (**B**) after cells were inoculated in prostate; (**C**) bioluminescence imaging of C4-2 3R orthotopic tumors weekly; (**D**) semi-quantification of total photon flux of bioluminescence imaging in the orthotopic tumor model; (**E**) T2-weighted image for the tumor-bearing mouse (#1) at fifth week. The tumor position was indicated by the dotted circle. B: bladder; asterisk: prostate; (**F**) bioluminescence imaging of C4-2 3R subcutaneous xenograft tumors weekly; (**G**) semi-quantification of total photon flux of bioluminescence imaging in the subcutaneous xenograft tumor model.

C4-2 cells are tumorigenic in immunodeficient male nude mice as reported by ATCC (https://www.atcc.org/products/crl-3314#detailed-product-information, accessed on 11 April 2024). To further characterize the tumor-forming capacity of C4-2 3R cells in vivo, we performed an orthotopic inoculation of C4-2 3R cells (Figure 2A). The surgical procedure of intraprostatic injection of C4-2 3R cells was documented by photos (Supplementary Figure S2). Representative photographs were also obtained to demonstrate the location of the tumor injection within the prostate. (Figure 2B). To monitor the tumor growth kinetics, bioluminescence imaging was performed weekly to track changes in photon flux from orthotopic C4-2 3R tumors. Bioluminescent signals of tumor-bearing mice gradually increased over the five-week observation period, although signal intensity varied among individual mice (Figure 2C). Semi-quantitative analysis of photon flux further confirmed the progressive increase in tumor growth (Figure 2D). Additionally, 7T µMRI using a T2 weighted sequence was performed to validate the bioluminescent signal of an orthotopic tumor at week 5 (Figure 2E). For comparison, a subcutaneous xenograft model was established using C4-2 3R cells. Although bioluminescent signals were detected in mice one week after inoculation, the signals were not sustained or propagated during the subsequent 5-week observation period (Figure 2F,G). No palpable and visible subcutaneous xenograft tumors detected after up to 8 weeks of follow-up.

### 3.3. Assessment of PSMA Expression After X-Ray Irradiation in C4-2 Cells

**Figure 3:**
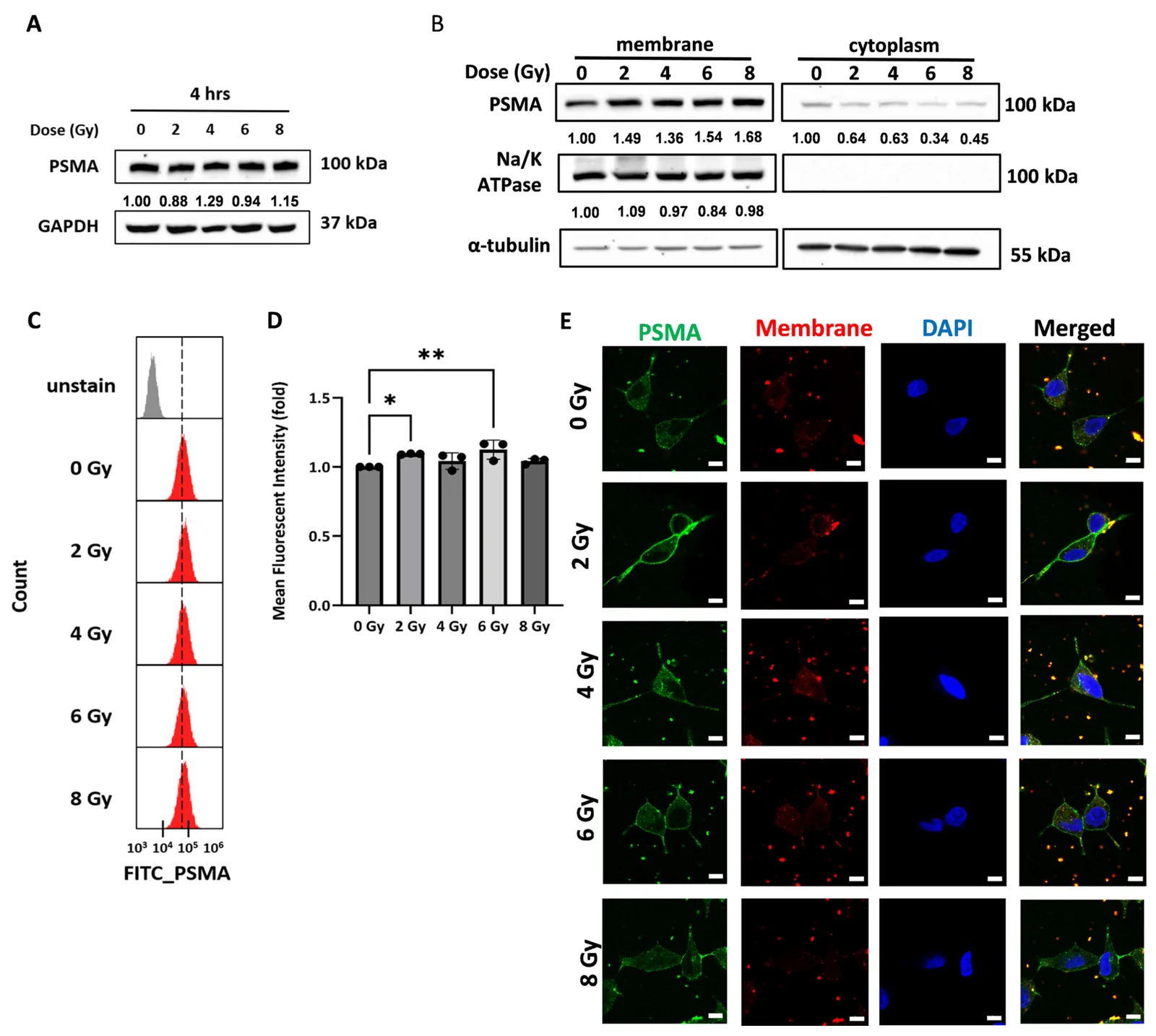
X-ray irradiation induced membrane PSMA in C4-2 cells. (**A**) Western blot analysis of total PSMA in C4-2 cells exposed to different doses or X-rays; (**B**) analysis of PSMA expression in cell membrane and cytoplasm; (**C**) flow cytometric analysis of PSMA on cell surface; (**D**) quantification of PSMA signals from the flow cytometry; (**E**) immunofluorescence imaging of PSMA expression 4 h after C4-2 cells exposed to different doses of X-rays. Scale bar: 10 µm. *: *p* < 0.05; **: *p* < 0.01.

It has been reported that X-ray EBRT can temporarily up-regulate PSMA expression in LNCaP cells [19]. Although C4-2 cells are derived from LNCaP subcutaneous xenograft tumor, they have been reported to exhibit greater radioresistance through increased expression of genes associated with cell cycle arrest and DNA repair [30]. Therefore, it remained important to determine the radiation response of PSMA in C4-2 cells, as this may influence the application of C4-2 3R cells for PSMA-targeting radioligand therapy and combination treatment in the orthotopic tumor model. C4-2 cells were first exposed to 2–8 Gy of X-ray irradiation to examine total PSMA protein expression. No detectable changes in total PSMA protein levels were observed 4 h after exposure (Figure 3A). As PSMA is a membrane protein, cell membrane was isolated to evaluate radiation-induced changes in PSMA after 4 h of irradiation. The results showed that membrane PSMA was equivalently increased after 2–8 Gy of exposure and was accompanied by a decrease in cytoplasmic PSMA compared with un-irradiated cells, although little change in cytoplasmic PSMA was observed after 4 and 8 Gy irradiation (Figure 3B). Membrane PSMA expression was further evaluated using flow cytometry, which showed a trend toward increased membrane PSMA following irradiation (Figure 3C). Quantitative analysis revealed that a non-dose dependent expression of membrane PSMA exhibited significant induction after 2 and 6 Gy irradiation compared with un-irradiated cells (Figure 3D). Moreover, fluorescent microscopy was used to visualize the membrane PSMA, which could be detected in cells exposed to X-rays, particularly at 2–6 Gy, whereas it was less apparent in un-irradiated cells (Figure 3E). Overall, these findings suggest that ionizing radiation promotes the redistribution of PSMA to the cell membrane rather than increasing total PSMA expression in C4-2 cells.

### 3.4. Potential of C4-2 3R Cells on Evaluation of Combined X-Ray EBRT and ^177^Lu-PSMA-617 Radioligand Therapy In Vivo

**Figure 4:**
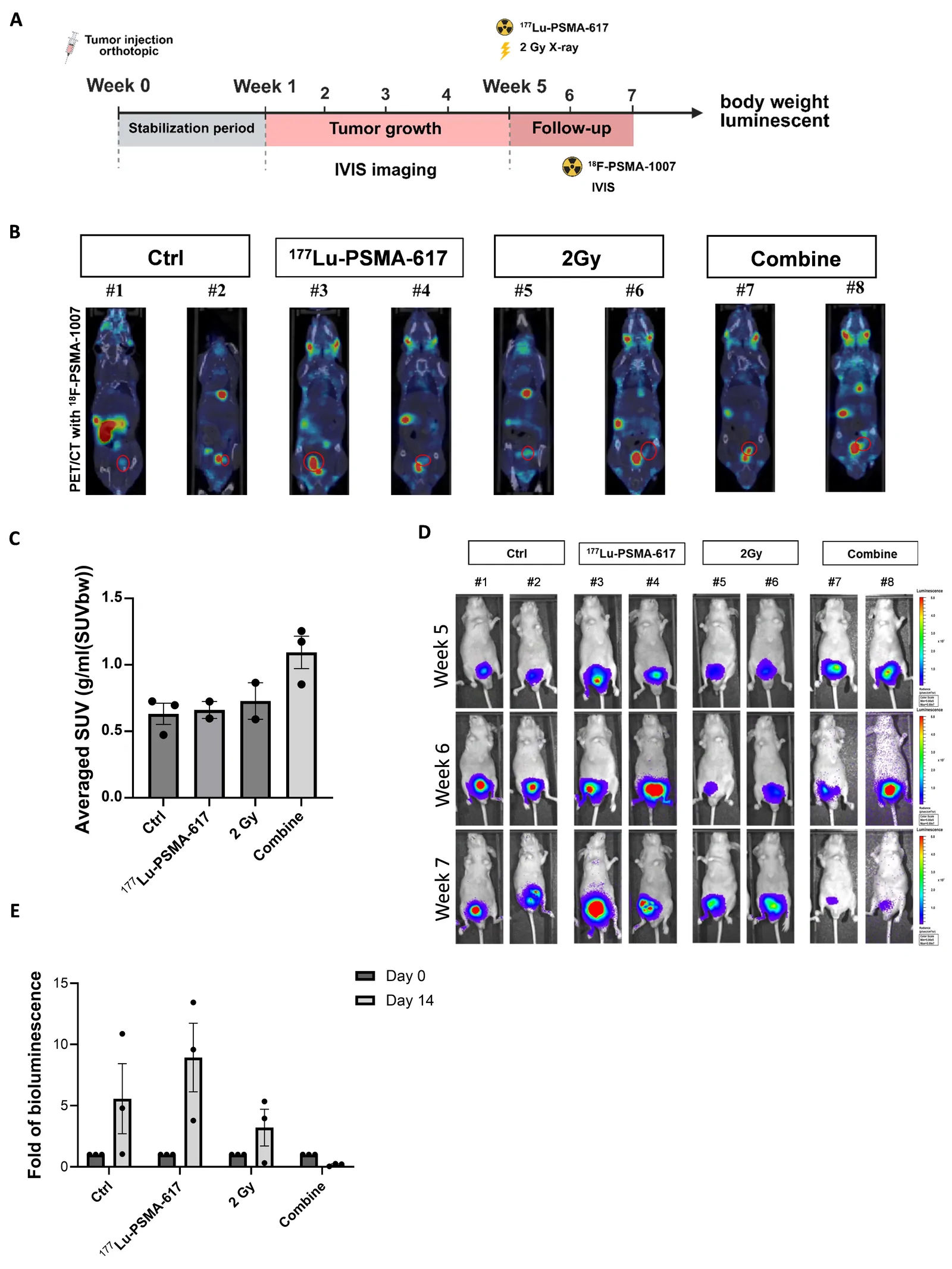
C4-2 3R orthotopic tumor model for monitoring the tumor responses to X-rays and ^177^Lu-PSMA-617 treatments. (**A**) Orthotopic tumor model for examination of tumor responses to different treatments using molecular imaging; (**B**) PET/CT imaging of tumor uptake for ^18^F-PSMA-1007 tracer after different treatments. The red circles indicate the regions of interest (ROIs) used for SUV analysis; (**C**) quantification of average SUV in PET/CT imaging, and analyzed by non-parametric one-way ANOVA; (**D**) bioluminescence imaging of tumor responses to different treatments; (**E**) semi-quantification of photon signals in bioluminescence imaging, and analyzed by two-way ANOVA with Tukey’s test. Ctrl: untreated control.

The use of the C4-2 3R cells to establish orthotopic prostate tumor model for therapeutic assessment of EBRT and ^177^Lu-PSMA-617 is shown in Figure 4A. After tumors were formed for 5 weeks, tumor-bearing mice received monotherapy or combination treatment with 2 Gy X-ray irradiation and ^177^Lu-PSMA-617. One week after treatment, μPET/CT imaging using ^18^F-PSMA-1007 was first performed to evaluate PSMA expression in orthotopic tumors (Figure 4B). The μPET/CT images were further quantified for preliminary comparison among treatment groups (Figure 4C). Notably, the analysis results were obtained without the baseline pre-treatment of ^18^F-PSMA-1007 imaging as a reference. Tumor responses to different treatments were monitored by bioluminescence imaging for two weeks. The results showed that photon signals from orthotopic tumors tended to decrease after combined X-ray irradiation and ^177^Lu-PSMA-617 treatment compared to either monotherapy (Figure 4D). Photon flux was semi-quantified for each treatment group between weeks 5 and 7 (Day 0 and 14 of treatments, respectively). The combination therapy demonstrated a trend toward treater tumor suppression of bioluminescent signals compared with ^177^Lu-PSMA-617 monotherapy (Figure 4E). Although the sample size of each therapeutic group was small and lacked sufficient statistical power, these results still demonstrated the potential of C4-2 3R cells for establishing a trackable orthoptic prostate tumor model to evaluate the therapeutic efficacy.

### 3.5. Dissection of C4-2 3R Cells Formed Orthotopic Tumor Tissue with Different Treatments

**Figure 5:**
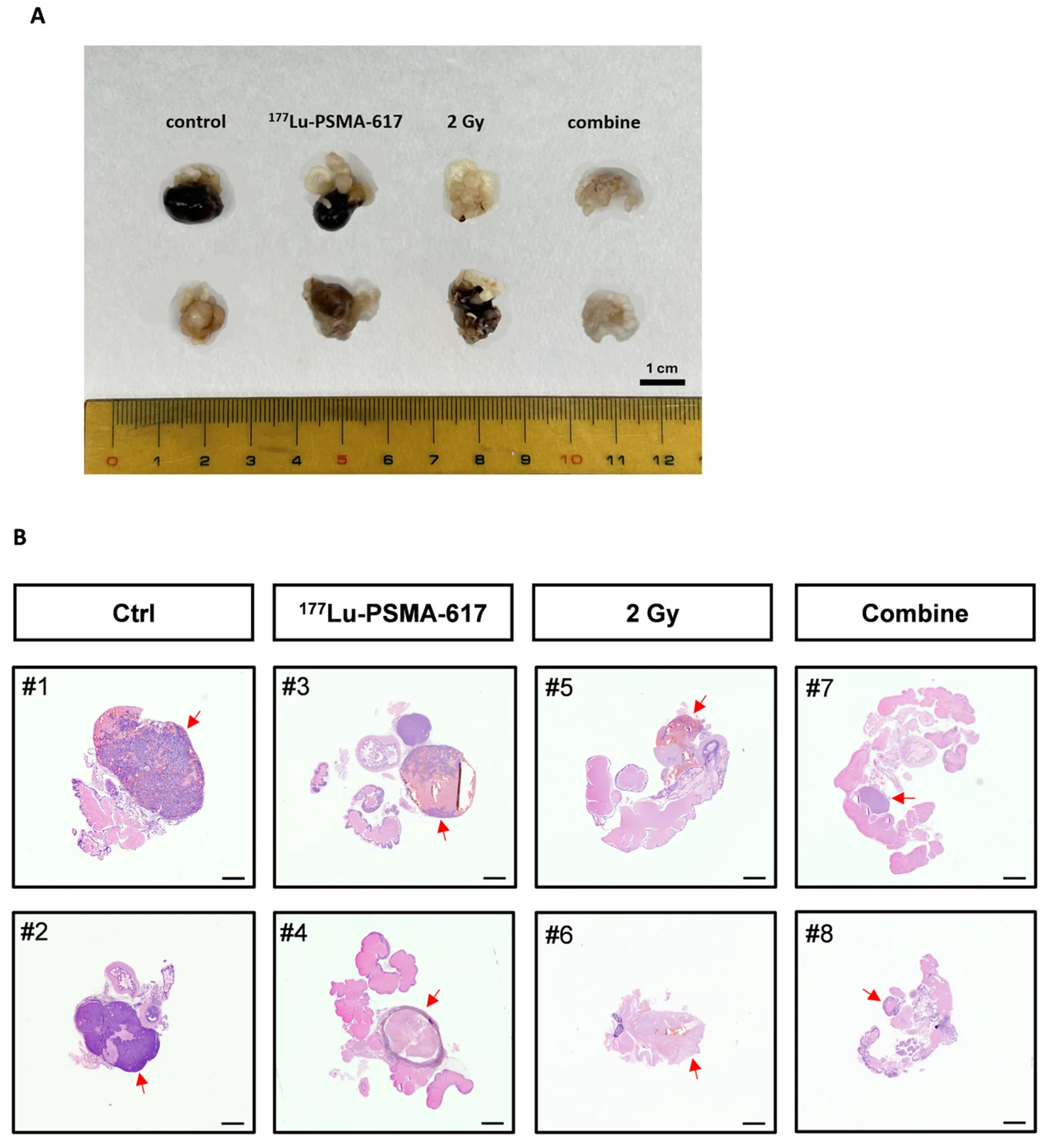
Dissection of C4-2 3R cells formed tumors from orthotopic prostate tumor model. (**A**) Orthotopic tumors excised from tumor-bearing mice after different treatments for 5 weeks. The numbers represent the mice indicated in Figure 4. Scale bar: 1 cm; (**B**) H & E staining of tumor sections from different treatments. The tumor positions were indicated by arrows. #1–#8 represented individual prostate samples from different mice. Ctrl: untreated control. Scale bar: 2000 µm.

After the final imaging acquisition, tumors from the prostate of tumor-bearing mice receiving different treatments were dissected for comparison. Tumors derived from C4-2 3R cells in the combination treatment group appeared substantially smaller than those in the monotherapy and control groups (Figure 5A). The dissected tumor tissues were further processed for histological sectioning and examination. Extensive staining of tumor tissues were mainly observed in the untreated control group, whereas different treatment regimes resulted in various levels of reduced tumor staining (Figure 5B).

## 4. Discussion

The slow-growing nature of PCa translates into correspondingly slow tumor development in xenograft models. LNCaP cells are commonly used to establish subcutaneous xenograft tumor model; however, palpable tumors usually require 2 to 3 months to develop, and tumor growth may remain limited or regress without continuous androgen supplementation [2,31]. In contrast, orthotopic prostate cancer models are considered to provide a more physiologically relevant organ-specific microenvironment for tumor development, although the technical complexity remains a major limitation for the model establishment [32,33]. Microscope-guided orthotopic injection of LNCaP cells has been reported to improve tumor establishment, but this approach requires detailed anatomical knowledge of the mouse prostate, appropriate surgical techniques, and specialized instruments [34]. Additionally, orthotopic tumor growth is generally monitored by ultrasound (US) or MRI, which requires well-trained and experienced radiographers and operators for image acquisition and interpretation. Importantly, the unpredictable growth kinetics of slow-growing PCa models reflect clinical diagnostic challenges, in which limited biopsy sampling may underestimate tumor grade and result in postoperative upgrading. Extended-core biopsy has been shown to improve detection accuracy and reduce upgrading risk [35], highlighting the importance of sampling strategies in risk stratification.

In this study, we introduced RGI as a simple and user-friendly approach for monitoring tumor growth kinetics and therapeutic responses. C4-2 PCa cells are derived from LNCaP cells, but their application in orthotopic tumor model has not been previously reported, except for C4-2B cells, which were derived from C4-2 and exhibit greater androgen-independent growth and bone metastasis potential [36]. Using C4-2 3R cells, the tumor that develops could be monitor as early as one week after inoculation. In particular, bioluminescence imaging represents a functional imaging approach in which photon signals reflect viable tumor cells, in contrast to anatomical imaging such as US and MRI. Thus, this approach provides a convenient method to determine whether the implanted tumors have successfully developed for further study, or whether additional animal studies should be initiated earlier to avoid prolonged waiting periods. Although *luc2* expression in C4-2 3R cells was primarily used to demonstrate the feasibility of bioluminescence imaging for monitoring orthotopic PCa tumors, these cells also harbor *mRFP* and *HSV1-tk* that could be used for in vivo fluorescent imaging and radionuclide-based imaging, respectively [37]. The multimodality molecular imaging capability of C4-2 3R cells may contribute to the establishment of a noninvasive and real-time trackable orthotopic PCa model for more efficient and reliable preclinical theranostic evaluation.

Before adopting the *PiggyBac* transposon system, we referred to a previously established C4-2-luc cell model generated by viral transduction of triple reporter genes [38]. However, C4-2 cells did not survive the transduction, which might be attributed to their high sensitivity to polybrene [39]. In contrast, the *PiggyBac* transposon system offers several advantages over viral transduction, including a larger cargo capacity, a less time-consuming and labor-intensive procedure, and stable genomic integration even in cells with relatively low transfection efficiency [14,15,24,40]. To the best of our knowledge, this is the first study demonstrating that the *PiggyBac* transposon system can successfully deliver reporter genes to C4-2 cells that are not amenable to viral transduction.

PSMA is an important molecular target for the theranostic treatment of PCa. However, PSMA expression has been reported to be low or deficient in approximately 30% of patients with mCRPC [41]. Therefore, induction of PSMA expression may have important clinical implications. EBRT is a standard treatment for PCa, and a recent study demonstrated that EBRT can increase the PSMA gene expression and enhance the uptake of ^177^Lu-PSMA-617 in a LNCaP subcutaneous xenograft model, although the treatment responses showed high variability [19]. This study also found that the expression of membrane PSMA in C4-2 cells increased after irradiation, but the response was not dose-dependent over the 2-8 Gy range. The selection of a 2 Gy EBRT dose and a 4 h interval before ^177^Lu-PSMA-617 administration was based on the previous study, in which surface PSMA expression increased most clearly at 4 h after irradiation and combination treatment resulted in improved tumor control [19]. However, additional time-course and dose-dependent experiments will be necessary to determine the optimal treatment conditions and to better support future therapeutic development. Whereas the previous study used a subcutaneous xenograft tumor model, we further applied the orthotopic C4-2 3R tumor model to validate the increased PSMA levels by PET/CT imaging. However, direct translation of findings from prior in vitro experiments and subcutaneous xenograft models to the present orthotopic model should be interpreted with caution because tissue geometry and local tumor microenvironment may influence radiation responses. Future time-course analyses in C4-2 cells will be required to further optimize the treatment interval.

Mechanistically, we found that X-ray irradiation did not increase total PSMA expression in C4-2 cells, but instead promoted the redistribution of PSMA to the cell membrane. Induction of membrane PSMA expression has been reported to be associated with DNA double-strand damage, induced by radiation and topoisomerase-2 inhibitors in PCa cells [42]. Radiation exposure is well known to activate DNA damage response (DDR) signaling pathways, including ATM/ATR kinase activation and γH2AX formation [43,44]. Therefore, the observed increase in PSMA membrane relocalization represents a broader radiation-induced cellular stress response rather than direct transcriptional upregulation. Previous studies have also suggested that radiation-associated modulation of PSMA is linked to DNA damage signaling events [45]. Further studies correlating PSMA trafficking with established DDR markers will be necessary to clarify the underlying mechanism. Although C4-2 cells are derived from LNCaP cells, they have become androgen-independent [46]. In addition, C4-2 cells are more radioresistant than LNCaP cells by expressing a set of genes associated with cell cycle arrest and DNA repair [30]. Whether the DNA repair capacity of PCa cells influences radiation-induced redistribution of PSMA should be further investigated.

Although C4-2 cells, including C4-2-luc cells, have been used to evaluate various therapeutic approaches, they have been mainly used for subcutaneous xenograft [22,38,47,48,49]. However, this study was unable to establish subcutaneous xenograft tumors using C4-2 3R cells. After subcutaneous inoculation of parental C4-2 cells into nude mice, only one of five mice developed a palpable tumor after three months of observation. Because C4-2 3R cells exhibited a slower proliferation rate than parental C4-2 cells, this may have contributed to the reduced tumor growth in the subcutaneous xenograft model. Nevertheless, the tumor growth in the orthotopic model remained robust, as demonstrated by bioluminescent imaging. In addition, reporter gene integration and expression may theoretically affect cellular stress or immunogenicity to reduce the tumor formation rate [50]. Therefore, although host-mediated rejection cannot be excluded, the inability of subcutaneous tumor formation using C4-2 3R cells may be associated with intrinsic biological characteristics of this cell line. Evaluation in more profoundly immunodeficient mouse strains (e.g., NSG or NCG mice) will be necessary to further clarify the role of immunity in experimental tumor models [51].

In summary, we used a non-viral *PiggyBac* transposon system to establish a novel PCa cell model for multimodality reporter gene imaging. Using bioluminescence imaging as a demonstration, the present results suggest that C4-2 3R cells are suitable for establishing an orthotopic prostate tumor model that enables convenient and real-time tracking of tumor progression, thereby reducing the prolonged and unpredictable waiting period associated with the slow-growing nature of PCa cells.

## 5. Limitations

The primary limitations include (1) the critically small sample size in the therapeutic groups; (2) the lack of pre-treatment baseline PET/CT imaging for therapeutic evaluation, and (3) the low tumor formation rate. Among the 36 nude mice inoculated with C4-2 3R cells, only 12 successfully developed orthotopic tumors suitable for theranostic monitoring, resulting in an overall engraftment rate of 33%. Because C4-2 3R cells showed a lower proliferative rate than parental C4-2 cells, this may have contributed to the tumor formation efficiency in vivo. The small sample size also limited the statistical analysis, requiring the use of non-parametric methods. Therefore, the results should be interpreted as preliminary and exploratory rather definitive. Future studies with larger cohorts will be necessary to validate these observations. Notably, baseline ^18^F-PSMA-1007 μPET/CT imaging before treatment would provide a more accurate assessment of treatment-induced changes in tracer uptake following either monotherapy or combination therapy in orthotopic tumors. C4-2 3R cells provide a practical model for real-time bioluminescence imaging to quickly compare the tumor progression in different microenvironments.

## 6. Conclusions

We established C4-2 3R cells expressing triple reporter genes for multimodality molecular imaging of prostate tumor progression in vivo. In the orthotopic model, tumors derived from C4-2 3R cells could be readily monitored noninvasively using bioluminescence imaging, and their growth was more robust than that observed in subcutaneous xenograft tumors. The C4-2 3R tumor model also offers a proof-of-concept for quick and real-time evaluation of novel therapeutic strategies, such as combined EBRT and RLT by ^177^Lu-PSMA-617. Interestingly, ionizing radiation promoted the membrane redistribution of PSMA in C4-2 cells rather than up-regulation of PSMA gene reported in LNCaP cells. Further mechanistic studies will be necessary to clarify the effects of radiation on PSMA expression in both androgen-dependent and androgen-independent PCa cells. Because of the limited sample size in the therapeutic study, the present findings should be considered preliminary rather than definitive. Additionally, long-term toxicity studies will be necessary to further evaluate the potential of combining the treatment of ^177^Lu-PSMA-617 and EBRT using this reporter gene tumor model. Overall, the establishment of an orthotopic PCa tumor model using C4-2 3R cells provides a reliable platform for the preclinical evaluation of emerging therapeutic strategies.

## Data Availability

The original contributions presented in this study are included in the article. Further inquiries can be directed to the corresponding author.

## Supplementary Materials

The following supporting information can be downloaded at: https://www.mdpi.com/article/10.3390/pharmaceutics18081009/s1, Figure S1: Analytical HPLC chromatogram. HPLC analysis was performed using a Waters 600 controller equipped with a Waters 2998 photodiode array detector and a radioactivity detector; Figure S2: Selection of PiggyBac transposon system transfected C4-2 cells by mRFP reporter gene; Figure S3: The surgical procedure for inoculation of C4-2 3R cells into the prostate of a nude mouse.
