# Supplementary material for: A Preliminary Evaluation of 177Lu-PSMA-617-Based Targeted Radioligand Therapy with X-Ray Stimulated PSMA Relocation Using the PiggyBac Reporter-Gene-Engineered Orthotopic Prostate Tumor Model
Source: Pharmaceutics. 2026 Aug 14;18(8):1009. doi: 10.3390/pharmaceutics18081009 (PMC13516927; doi:10.3390/pharmaceutics18081009)
Supplement: Supplementary file 1 [file pharmaceutics-18-01009-s001.zip › pharmaceutics-4439222-supplementary.pdf]

pharmaceutics

Figure Legends for Supplementary

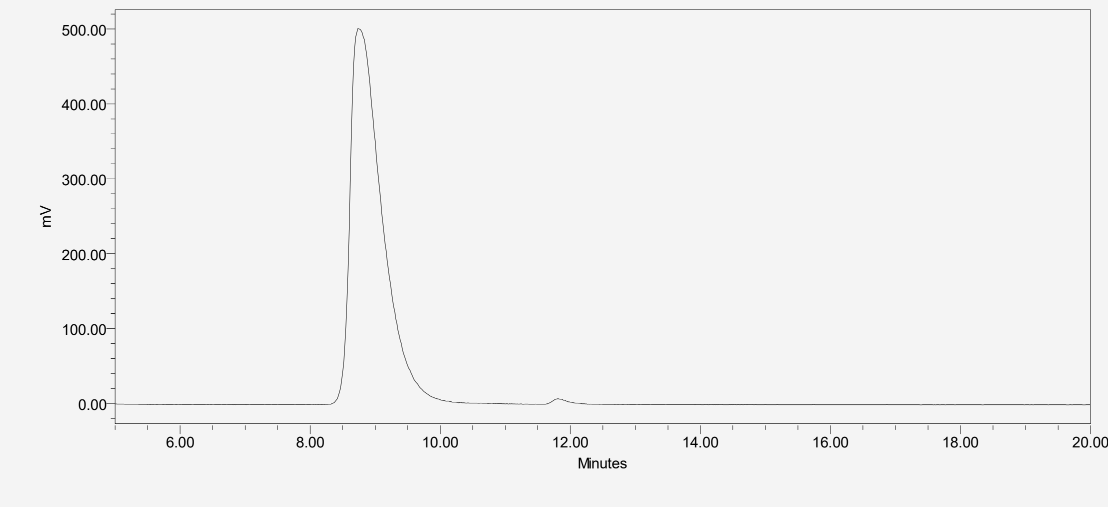

Supplementary Figure S1. Analytical HPLC chromatogram. HPLC analysis was performed using a Waters 600 controller equipped with a Waters 2998 photodiode array detector and a radioactivity detector (Flow Count FC-1000, BioScan). An analytical ReproSil 100 C18 column (Dr. Maisch) was used with a gradient mobile phase consisting of water containing 0.1% TFA (solvent A) and acetonitrile containing 0.1% TFA (solvent B). The gradient was as follows: 95% A/5% B at 0 min, 20% A/80% B at 25 min, 5% A/95% B at 29 min, and a return to 95% A/5% B at 35 min. The flow rate was 1 mL/min.

 https://doi.org/10.3390/pharmaceutics18081009

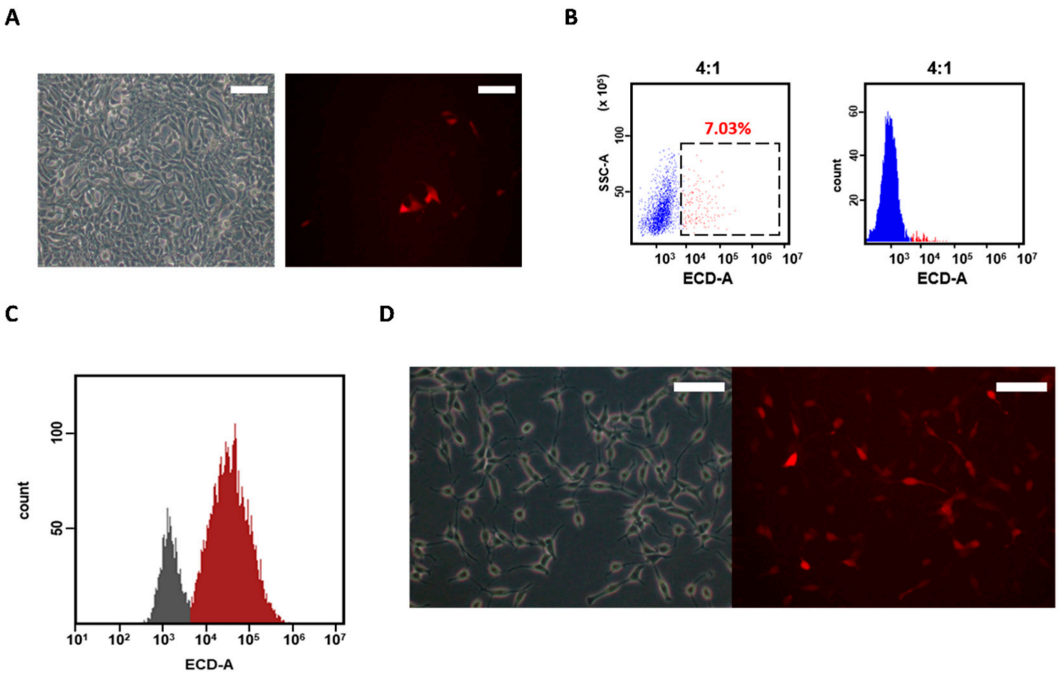

Supplementary Figure S2. Selection of *PiggyBac* transposon system transfected C4-2 cells by mRFP reporter gene. (A) Visualization of mRFP expressing cells after the first round of cell co-transfection with *PiggyBac* reporter construct and PBase construct (4:1). (B) Flow cytometric quantification of mRFP expressing cells. (C) FACS sorting of mRFP expressing cells. (D) Visualization of mRFP expressing cells after cell sorting.

https://doi.org/10.3390/pharmaceutics18081009

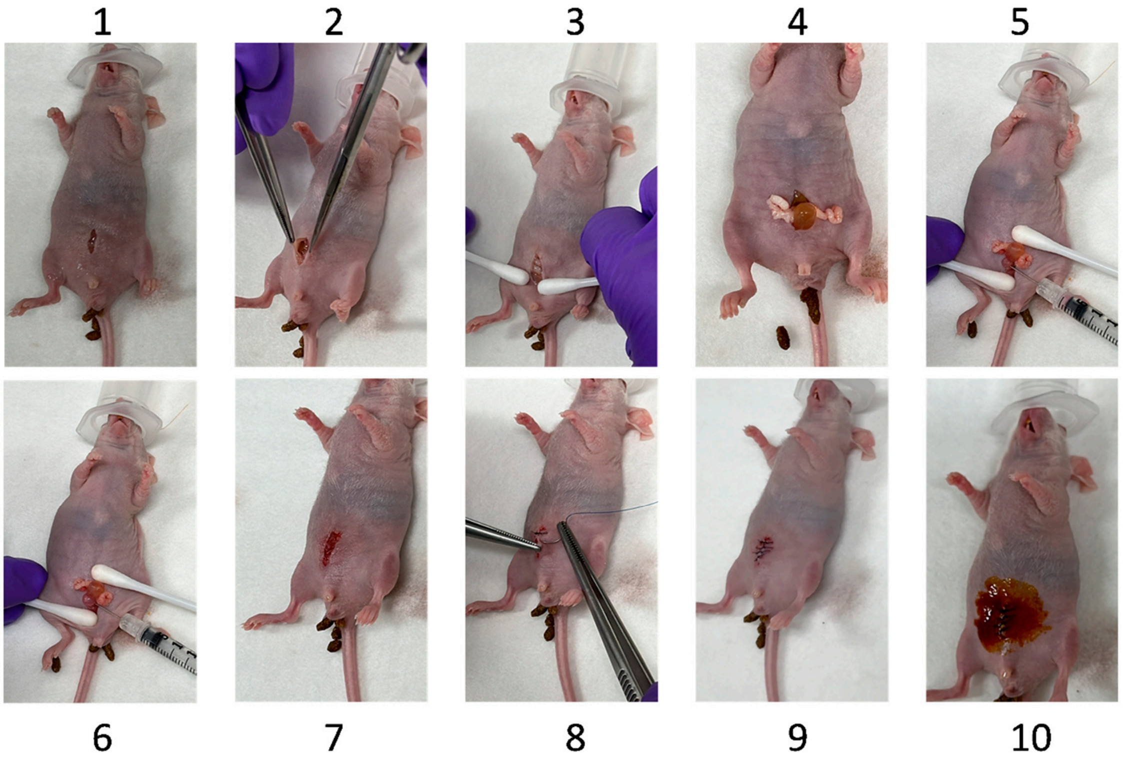

Supplementary Figure S3. The surgical procedure for inoculation of C4-2 3R cells into the prostate of a nude mouse. 1. Disinfection of the lower abdominal area using 70% ethanol and make a 0.5-cm skin incision. 2. Use of teeth forceps and scissors to carefully open the underlying muscle layer. 3. Gently externalize the bladder using a sterile cotton swab. 4. Gently pull out the seminal vesicle adjacent to the bladder. 5. Slowly injection of 20 µL of tumor cell suspension into the prostate. 6. After a short pause, withdraw the needle and ensure that no leakage of the cell suspension occurs. 7. Close the muscle layer using chromic catgut absorbable sutures. 8. Close the skin using monofilament nylon non-absorbable sutures. 9. Confirm that the suturing is completed properly. 10. Disinfect the incision area with povidone-iodine.

https://doi.org/10.3390/pharmaceutics18081009
